# Incidence and variability in receipt of phenotype-desirable antimicrobial therapy for Enterobacterales bloodstream infections among hospitalized United States patients

**DOI:** 10.1017/ash.2024.444

**Published:** 2024-10-22

**Authors:** Rena C. Moon, Shawn H. MacVane, Joy David, Jacob B. Morton, Ning Rosenthal, Kimberly C. Claeys

**Affiliations:** 1 PINC AI Applied Sciences, Premier Inc., Charlotte, NC, USA; 2 Global Medical Affairs, bioMérieux, Salt Lake City, UT, USA; 3 Global Marketing, bioMérieux, Salt Lake City, UT, USA; 4 School of Pharmacy, University of Maryland, Baltimore, MD, USA

## Abstract

**Background:**

Using a large, geographically diverse, hospital-based database in the United States (Premier PINC AI Healthcare Database), we aimed to describe the proportion and characteristics of patients receiving phenotype-desirable antimicrobial therapy (PDAT) among those hospitalized with Enterobacterales bloodstream infections.

**Methods:**

Adult patients with an admission between January 1, 2017 and June 30, 2022 with ≥1 blood culture positive for *Escherichia coli*, *Klebsiella oxytoca*, *Klebsiella pneumoniae*, or *Proteus mirabilis* and receiving an empiric antibiotic therapy on blood culture collection (BCC) Days 0 or 1 were included. Receiving PDAT (defined as receipt of any antimicrobial categorized as “desirable” for the respective phenotype) on BCC Days 0−2 was defined as receiving early PDAT.

**Results:**

Among 35,880 eligible patients, the proportion of patients receiving PDAT increased (from 6.8% to 22.8%) from BCC Day 0−4. Patients who received PDAT (8,193, 22.8%) were more likely to visit large (500 + beds, 36% vs 31%), teaching (45% vs 39%), and urban (85% vs 82%) hospitals in the Northeast (22% vs 13%) compared to patients not receiving PDAT (all *P* <. 01). Among patients receiving PDAT, 61.4% (n = 5,033) received it early; they had a lower mean comorbidity score (3.2 vs 3.6), were less likely to have severe or extreme severity of illness (71% vs 79%), and were less likely to have a pathogen susceptible to narrow-spectrum β-lactams (31% vs 71%) compared to patients in the delayed PDAT group (all *P* < .01).

**Conclusions:**

The proportion of patients receiving desirable therapy increased between BCC Day 0 and 4. Receipts of PDAT and early PDAT were associated with hospital, clinical, and pathogen characteristics.

## Introduction

Gram-negative (GN) bloodstream infection (BSI) is associated with high morbidity and mortality among hospitalized patients.^1^ The emergence of multi-drug resistant pathogens makes the treatment of GN-BSI more challenging and therefore identifying the causative pathogen and testing its antimicrobial susceptibility is fundamental to optimal managment.^2^ Initiating effective therapy early is significantly associated with improved survival among patients hospitalized with GN-BSI.^3,4^ To increase the likelihood of providing effective therapy, broad-spectrum empiric antimicrobial regimens are often prescribed to patients while awaiting results of microbiological testing. However, overly broad therapy, or overtreatment, is also considered an inappropriate therapy since it is associated with selective pressure for antibiotic resistance and unnecessary adverse events. Inappropriate antimicrobial use is one of the reasons for the emergence of antimicrobial-resistant bacteria^5−8^; the use of narrow-spectrum agents and targeted therapy are preferable as long as patient outcomes are not adversely affected.^9−11^

Although rapid diagnostic testing (RDT) has been used to tailor therapy, RDTs are supplemental tests. Tailoring therapy is often delayed until final susceptibilities are provided and most RDTs do not provide phenotypic susceptibility information. Furthermore, RDTs for de-escalation remain poorly defined as broader variety of resistance mechanisms are not completely captured by existing RDTs.^12,13^

Phenotype-desirable antimicrobial therapy (PDAT, defined as receipt of any antimicrobial categorized as “desirable” for the respective phenotype) is crucial for antimicrobial stewardship as providing appropriate targeted therapy early can prevent the emergence of antimicrobial-resistant bacteria and *Clostridioides difficile* and prevent antimicrobial-associated adverse drug events.^14–16^ However, timing of targeted therapy is not well understood, nor are characteristics and types of patients receiving early PDAT (within 2 days of blood culture collection [BCC]).

The main objectives of this study are to 1) estimate the proportion of patients receiving PDAT, and early PDAT, 2) describe and compare the patient demographics, hospital characteristics, and clinical characteristics of patients receiving PDAT versus not receiving PDAT, and 3) describe treatment patterns of patients receiving early PDAT and delayed PDAT among those hospitalized with *Escherichia coli*, *Klebsiella oxytoca*, *Klebsiella pneumoniae*, or *Proteus mirabilis* BSI.

## Methods

### Data source and study design

We performed a retrospective observational cohort study using the Premier PINC AI Healthcare Database (PHD).^17^ The PHD is an all-payer hospital administrative database for geographically diverse inpatient and outpatient visits from more than 1,300 hospitals. Inpatient discharges in PHD represent approximately 25% of all inpatient admissions in the US since 2000. PHD patients are tracked within the same hospital or hospital system using a unique identifier.^17,18^ The standard hospital discharge files include demographic characteristics, disease states, and a time-stamped log of billed items (e.g., procedures, medications, laboratory services, and diagnostic services) at the patient level, and geographic location, rural/urban populations served, teaching status, and bed capacity at the hospital-level. A subset of hospitals (∼25%) submit microbiology laboratory data to the PHD. Microbiology data include specimen collection date, type of specimen, types of tests performed, and observations for the specimens. All data are statistically deidentified and compliant with the Health Insurance Portability and Accountability Act. Based on US Title 45 Code of Federal Regulations, Part 46, the study was exempted from institutional review board approval. We did not pursue informed consent of study participants due to the nature of deidentified data. The study followed the Strengthening the Reporting of Observational Studies in Epidemiology (STROBE)^19^ reporting guideline.

### Study population

Adults (aged ≥ 18 years) who had an inpatient visit, had ≥ 1 blood culture isolate belonging to *E. coli*, *K. oxytoca*, *K. pneumoniae*, or *P. mirabilis*, and received empiric IV or oral β-lactam antimicrobial therapy (within 2 days of BCC) between January 1, 2017, and June 30, 2022, were included in the study. Patients were excluded if they: (1) had polymicrobial infection defined as positive cultures belonging to > 1 pathogens from blood or any other site within 30 days before and 5 days after the index BCC date, (2) did not have antimicrobial susceptibility testing (AST) results available by BCC Day 7, (3) had insufficient AST results to define susceptibility profile, (4) did not receive systemic antimicrobial treatment of β-lactam or other oral (fluoroquinolone [FQ] or trimethoprim-sulfamethoxazole [TMP-SMX]) agents for at least 3 consecutive days on or after BCC Day 0, (5) had an intra-abdominal infection (to limit polymicrobial infections and because treatment for intra-abdominal infections may require coverage for additional organisms)^20^, (6) were transferred from another acute care facility, (7) expired, discharged, or transferred to another hospital within 2 days following BCC Day 0, and (8) were from hospitals without continuous data submission during the 3 months before and 30 days after the visit. If a patient had multiple hospitalizations meeting the selection criteria, the earliest admission meeting all inclusion and exclusion criteria was considered as “index admission”.

### Definitions of DOOR-MAT and PDAT

**Figure 1.**
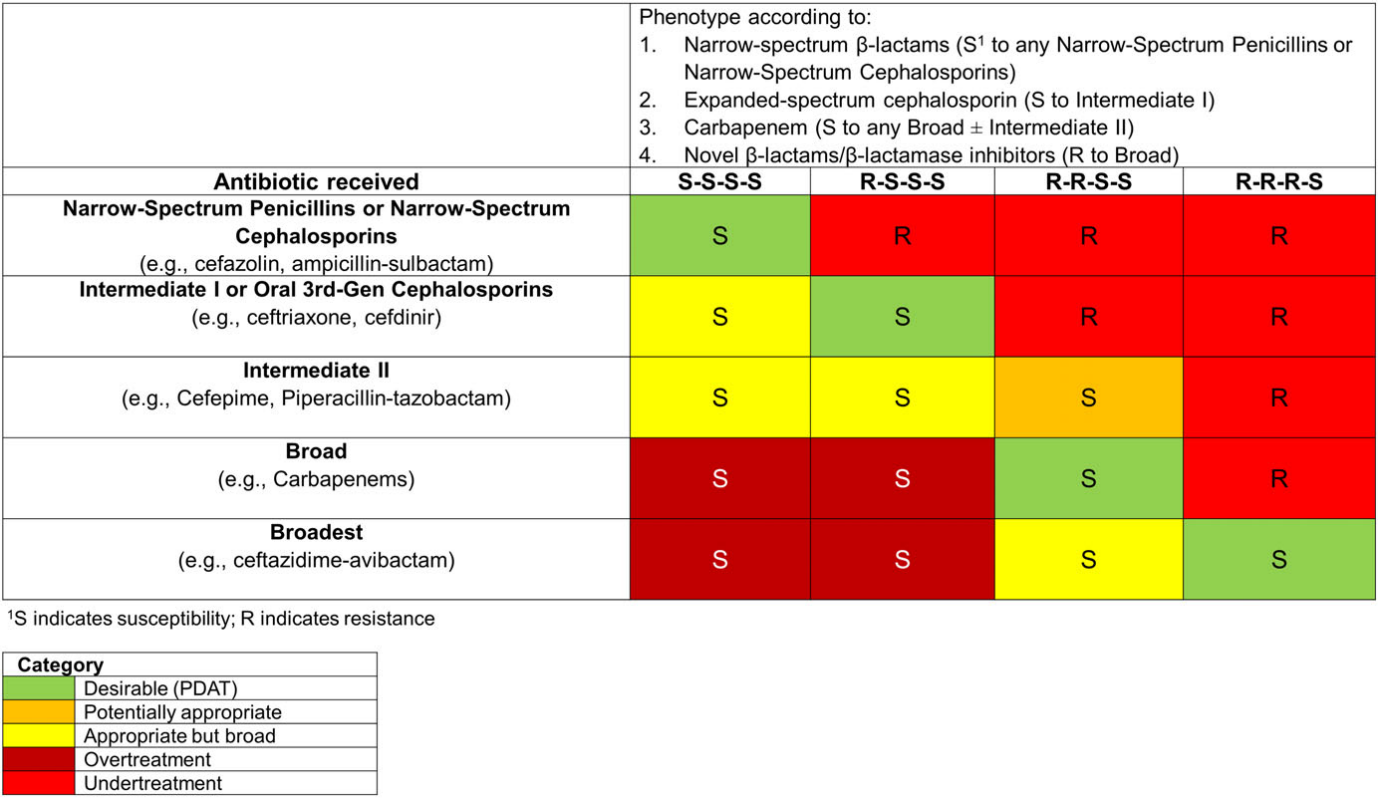
Grid illustrating the desirability of outcome ranking for the management of antimicrobial therapy (DOOR-MAT) for the treatment of bloodstream infections caused by *Escherichia coli, Klebsiella oxytoca, Klebsiella pneumoniae,* or *Proteus mirabilis*.

PDAT was the primary variable of interest in this study and defined as the β-lactam antibiotic with the narrowest spectrum that is *in vitro* active against the final isolated pathogen. This is operationalized and denoted using the Desirability of Outcome Ranking for the Management of Antimicrobial Therapy (DOOR-MAT) framework (Figure 1). The definition of DOOR-MAT categories was adapted from the naïve approach in the paper by Perez *et al.* ^20^ Antimicrobials were classified by their spectrum of activity (Supplement eTable 1) with a focus on β-lactams as these are preferred for BSI. Use of an antimicrobial inactive against a bacterial phenotype was considered as “undertreatment.” Use of an Intermediate II antimicrobial (e.g., piperacillin-tazobactam) for isolates that were resistant to expanded-spectrum cephalosporins (e.g., ceftriaxone) was defined as “potentially appropriate”, but not as receiving desirable treatment (PDAT), given recent evidence that carbapenems may be most desirable for this phenotype.^21^ Among patients receiving PDAT, patients were identified as ‘receiving early PDAT’ if desirable treatment was given within 2 days of BCC (Day 0−2), and ‘receiving delayed PDAT’ otherwise.

In a subgroup of patients who received oral antimicrobials, a secondary analysis was performed wherein oral fluoroquinolone (FQ), trimethoprim-sulfamethoxazole (TMP-SMX), or β-lactams within 4 days of BCC (Day 0–4) were defined as effective oral therapy if the pathogen was *in vitro* susceptible. Oral agents were not included in defining PDAT by DOOR-MAT because (1) these could not be ranked by spectrum of activity within context of β-lactams, (2) there remains controversy regarding the appropriate use of oral β-lactams for GN-BSI, and (3) for oral TMP-SMX and FQ, these are often deemed preferred to transition to oral therapy in GN-BSI.

### Patient, hospital, and visit characteristics

Baseline patient characteristics and hospital characteristics including geographical region (i.e., Midwest, Northeast, South, or West), hospital size, urbanicity of served population (rural vs urban), and teaching status were provided by the hospitals. Charlson-Deyo comorbidities were identified during index visit and any visit to the same hospital within 6 months prior to the index visit using International Classification of Diseases, 10th Revision, Clinical Modification (ICD-10-CM) and International Classification of Diseases, 10th Revision, Procedural Coding System (ICD-10-PCS) codes (Supplement eTable 2) and Charlson-Deyo Comorbidity Index (CCI) score was calculated using a previously validated method.^22,23^ The 3M All Patient Refined Diagnosis Related Groups (APR-DRG) classification of Severity of Illness (SOI) and Risk of Mortality (ROM) measures were used. APR-DRG SOI and ROM are a previously validated method of estimating the extent of physiologic decompensation and the likelihood of in-hospital death using four subclass levels (minor, moderate, major, and extreme).^24,25^

DOOR-MAT agents and oral agents were identified using hospital chargemaster descriptions. Time to first AST results was calculated by subtracting the time of BCC from the time of result observation. All AST results were available as S (susceptible), I (intermediate), or R (resistant).

### Statistical analysis

Descriptive statistics were used to present baseline patient and hospital characteristics of GN-BSI patients and their outcomes. Continuous variables were reported as mean (standard deviation) or median (1^st^ quartile, 3^rd^ quartile), and categorical variables were reported as counts and percentages. For statistical difference between two groups, the Student’s t-test or Mann–Whitney test was used for continuous variables, as indicated, and Pearson’s χ^2^ test or Fisher’s exact test for categorical variables. All analyses and figures were performed and generated using R v.3.6.3 or higher (R Foundation for Statistical Computing, Vienna, Austria).

## Results

**Figure 2.**
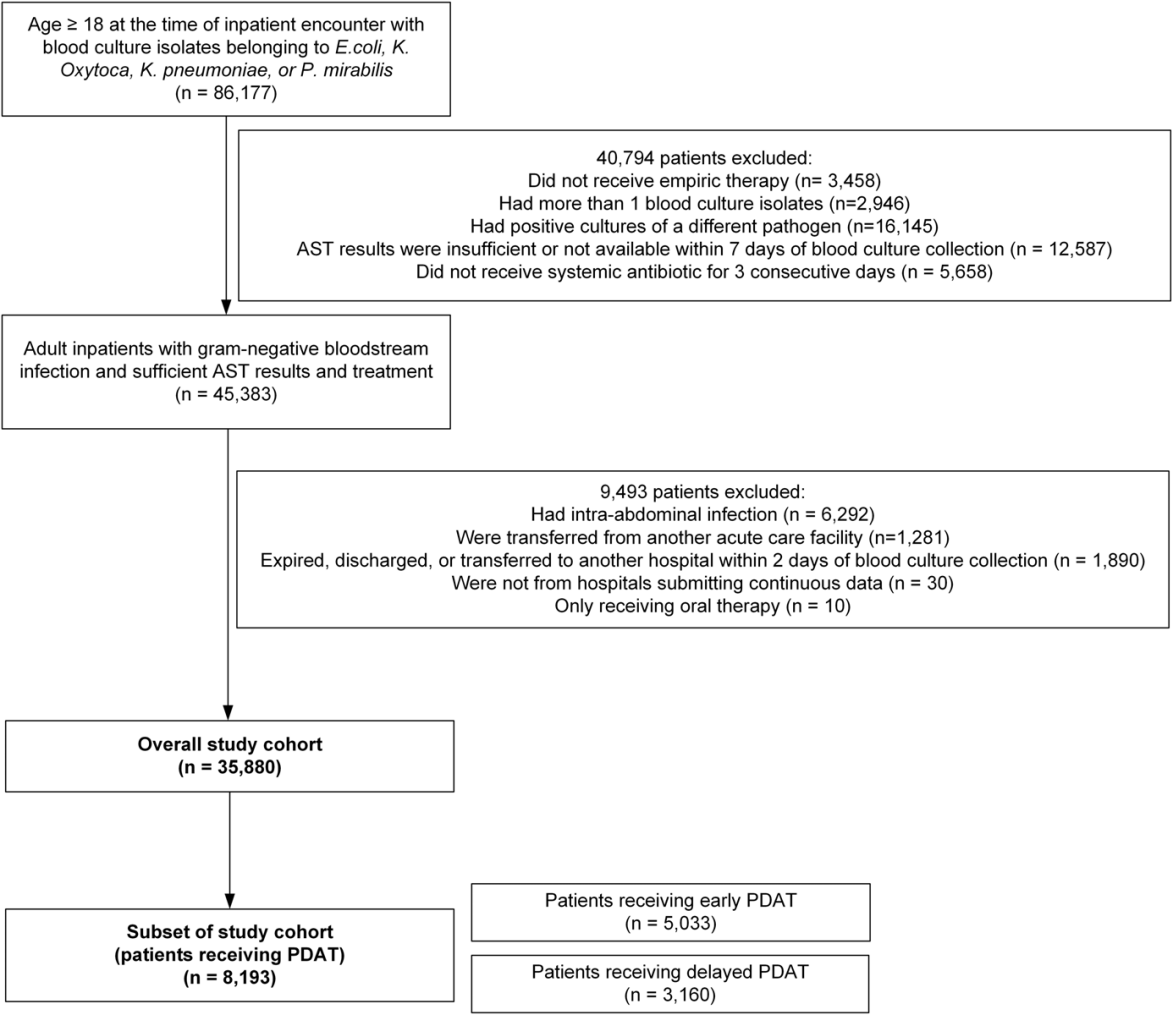
Flowchart of patient selection.

We identified 35,880 hospitalized adult patients meeting the study selection criteria (Figure 2). Patient characteristics are shown in Table 1. Approximately 23% of these patients (n = 8,193) received PDAT and 12% (n = 4,155) patients received effective oral therapy.

**Table 1.** Demographic, hospital, and visit and clinical characteristics of patients hospitalized with gram-negative bloodstream infection, stratified by phenotype-desirable antimicrobial therapy (PDAT) status

	Overall		PDAT		Non-PDAT		*P* values
	(n = 35,880)		(n = 8,193)		(n = 27,687)		
***Patient demographics***							
**Age, continuous, in years**							
Mean-Std Dev	68.7	16.5	69.0	16.4	68.6	16.5	*0.04*
**Sex, n (%)**							
Male	14,526	40.5%	3,435	41.9%	11,091	40.1%	*0.01*
Female	21,353	59.5%	4,758	58.1%	16,595	59.9%	
Unknown	1	0.0%	0	0.0%	1	0.0%	
**Race, n (%)**							
White	26,551	74.0%	5,778	70.5%	20,773	75.0%	*<0.01*
Black	4,769	13.3%	1,200	14.6%	3,569	12.9%	
Asian	1,495	4.2%	396	4.8%	1,099	4.0%	
Other	2,488	6.9%	703	8.6%	1,785	6.4%	
Unknown	577	1.6%	116	1.4%	461	1.7%	
**Ethnicity, n (%)**							
Hispanic	3,066	8.5%	729	8.9%	2,337	8.4%	*<0.01*
Non-Hispanic	28,919	80.6%	6,393	78.0%	22,526	81.4%	
Unknown	3,895	10.9%	1,071	13.1%	2,824	10.2%	
**Insurance type, n (%)**							
Medicare	24,701	68.8%	5,746	70.1%	18,955	68.5%	*<0.01*
Medicaid	3,496	9.7%	870	10.6%	2,626	9.5%	
Commercial insurance	5,501	15.3%	1,140	13.9%	4,361	15.8%	
Uninsured	1,423	4.0%	273	3.3%	1,150	4.2%	
Other/Unknown	759	2.1%	164	2.0%	595	2.1%	
***Visit and Clinical Characteristics***							
**Charlson-Deyo comorbidities, n (%)**							
Myocardial infarction	4,950	13.8%	1,176	14.4%	3,774	13.6%	*0.10*
Congestive heart failure	8,702	24.3%	2,100	25.6%	6,602	23.8%	*<0.01*
Peripheral vascular disease	3,732	10.4%	859	10.5%	2,873	10.4%	*0.78*
Cerebrovascular disease	4,353	12.1%	1,023	12.5%	3,330	12.0%	*0.26*
Dementia	5,751	16.0%	1,491	18.2%	4,260	15.4%	*<0.01*
Chronic pulmonary disease	9,416	26.2%	2,161	26.4%	7,255	26.2%	*0.76*
Rheumatic disease	1,511	4.2%	352	4.3%	1,159	4.2%	*0.66*
Peptic ulcer disease	836	2.3%	185	2.3%	651	2.4%	*0.62*
Mild liver disease	808	2.3%	186	2.3%	622	2.2%	*0.90*
Diabetes	15,797	44.0%	3,697	45.1%	12,100	43.7%	*0.02*
Hemiplegia or paraplegia	872	2.4%	213	2.6%	659	2.4%	*0.26*
Renal disease	10,818	30.2%	2,627	32.1%	8,191	29.6%	*<0.01*
Moderate or severe liver disease	992	2.8%	211	2.6%	781	2.8%	*0.23*
Any malignancy	5,842	16.3%	1,288	15.7%	4,554	16.4%	*0.12*
Metastatic solid tumor	2,222	6.2%	444	5.4%	1,778	6.4%	*<0.01*
HIV disease	144	0.4%	36	0.4%	108	0.4%	*0.54*
**Charlson Comorbidity Index (CCI) score**							
Mean-Std Dev	3.27	2.85	3.32	2.83	3.25	2.86	*0.06*
Median (Q1–Q3)	3.00	(1.00–5.00)	3.00	(1.00–5.00)	3.00	(1.00–5.00)	
**Severity of illness (3M APR-DRG), n (%)**							
Minor	1,269	3.5%	221	2.7%	1,048	3.8%	*<0.01*
Moderate	8,853	24.7%	1,877	22.9%	6,976	25.2%	
Severe	15,052	42.0%	3,569	43.6%	11,483	41.5%	
Extreme	10,706	29.8%	2,526	30.8%	8,180	29.5%	
**Treatment patterns, n (%)**							
S-S-S-S	30,289	84.4%	3,811	46.5%	26,478	95.6%	*<0.01*
R-S-S-S	3,048	8.5%	2,323	28.4%	725	2.6%	
R-R-S-S	2,460	6.9%	2,055	25.1%	405	1.5%	
R-R-R-S	83	0.2%	4	0.0%	79	0.3%	
**Types of infection organism, n (%)**							
*E. coli*	25,591	71.3%	6,395	78.1%	19,196	69.3%	*<0.01*
*K. oxytoca*	641	1.8%	104	1.3%	537	1.9%	
*K. pneumoniae*	6,347	17.7%	1,124	13.7%	5,223	18.9%	
*P. mirabilis*	3,301	9.2%	570	7.0%	2,731	9.9%	
**Concurrent bacterial infection, present on admission, n (%)**							
Pneumonia	4,102	11.4%	809	9.9%	3,293	11.9%	*<0.01*
Urinary tract infection	28,331	79.0%	6,603	80.6%	21,728	78.5%	*<0.01*
Sepsis	29,727	82.9%	6,749	82.4%	22,978	83.0%	*0.19*
Bacteremia	3,534	9.8%	833	10.2%	2,701	9.8%	*0.27*
Central venous catheter-related infection	398	1.1%	76	0.9%	322	1.2%	*0.07*
**First effective antibiotic, n (%)** ^*^							
Narrow-Spectrum Penicillins (e.g., ampicillin, amoxicillin)	1,739	4.8%	1,362	16.6%	377	1.4%	*<0.01*
Narrow-Spectrum Cephalosporins (e.g., cefazolin, cephalexin)	3,289	9.2%	2,603	31.8%	686	2.5%	*<0.01*
Oral 3rd Generation Cephalosporins (e.g., cefixime, cefdinir)	962	2.7%	62	0.8%	900	3.3%	*<0.01*
Intermediate I (e.g., ceftriaxone, cefotaxime)	25,445	70.9%	4,731	57.7%	20,714	74.8%	*<0.01*
Intermediate II (e.g., cefepime, piperacillin-tazobactam)	24,119	67.2%	4,976	60.7%	19,143	69.1%	*<0.01*
Broad (e.g., ertapenem, doripenem)	8,883	24.8%	3,037	37.1%	5,846	21.1%	*<0.01*
Broadest (e.g., ceftazidime-avibactam)	7,151	19.9%	1,977	24.1%	5,174	18.7%	*<0.01*
Fluoroquinolones (FQ)	4,389	12.2%	566	6.9%	3,823	13.8%	*<0.01*
Trimethoprim-sulfamethoxazole (TMP-SMX)	579	1.6%	126	1.5%	453	1.6%	*0.53*
***Hospital characteristics***							
**Hospital Size, n (%)**							
1–299 beds	14,939	41.6%	3,214	39.2%	11,725	42.3%	*<0.01*
300–499 beds	9,370	26.1%	2,029	24.8%	7,341	26.5%	
500+beds	11,571	32.2%	2,950	36.0%	8,621	31.1%	
**Teaching Status, n (%)**							
Teaching hospital	14,574	40.6%	3,690	45.0%	10,884	39.3%	*<0.01*
Non-teaching hospital	21,306	59.4%	4,503	55.0%	16,803	60.7%	
**Population Served, n (%)**							
Rural	6,367	17.7%	1,266	15.5%	5,101	18.4%	*<0.01*
Urban	29,513	82.3%	6,927	84.5%	22,586	81.6%	
**Geographic Location, n (%)**							
Midwest	6,575	18.3%	1,146	14.0%	5,429	19.6%	*<0.01*
Northeast	5,382	15.0%	1,791	21.9%	3,591	13.0%	
South	22,087	61.6%	4,773	58.3%	17,314	62.5%	
West	1,836	5.1%	483	5.9%	1,353	4.9%	

APR-DRG, All Patient Defined Diagnosis Related Groups; HIV, human immunodeficiency virus.

* If the patient received more than one applicable agent on the same day, all given agents were captured.

**Figure 3.**
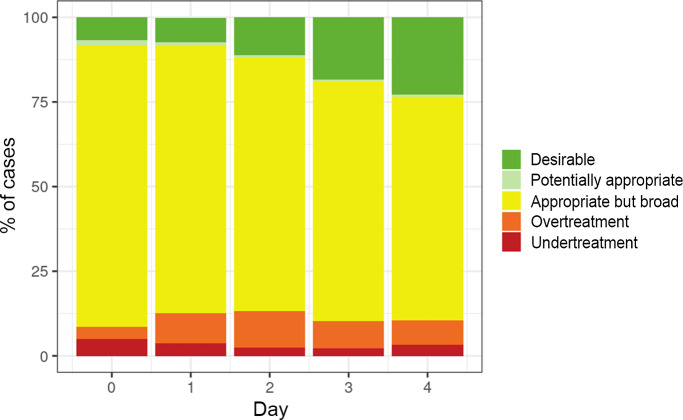
Proportion of patients receiving each desirability of outcome ranking for the management of antimicrobial therapy (DOOR-MAT) category agents between blood culture collection days 0–4.

**Figure 4.**
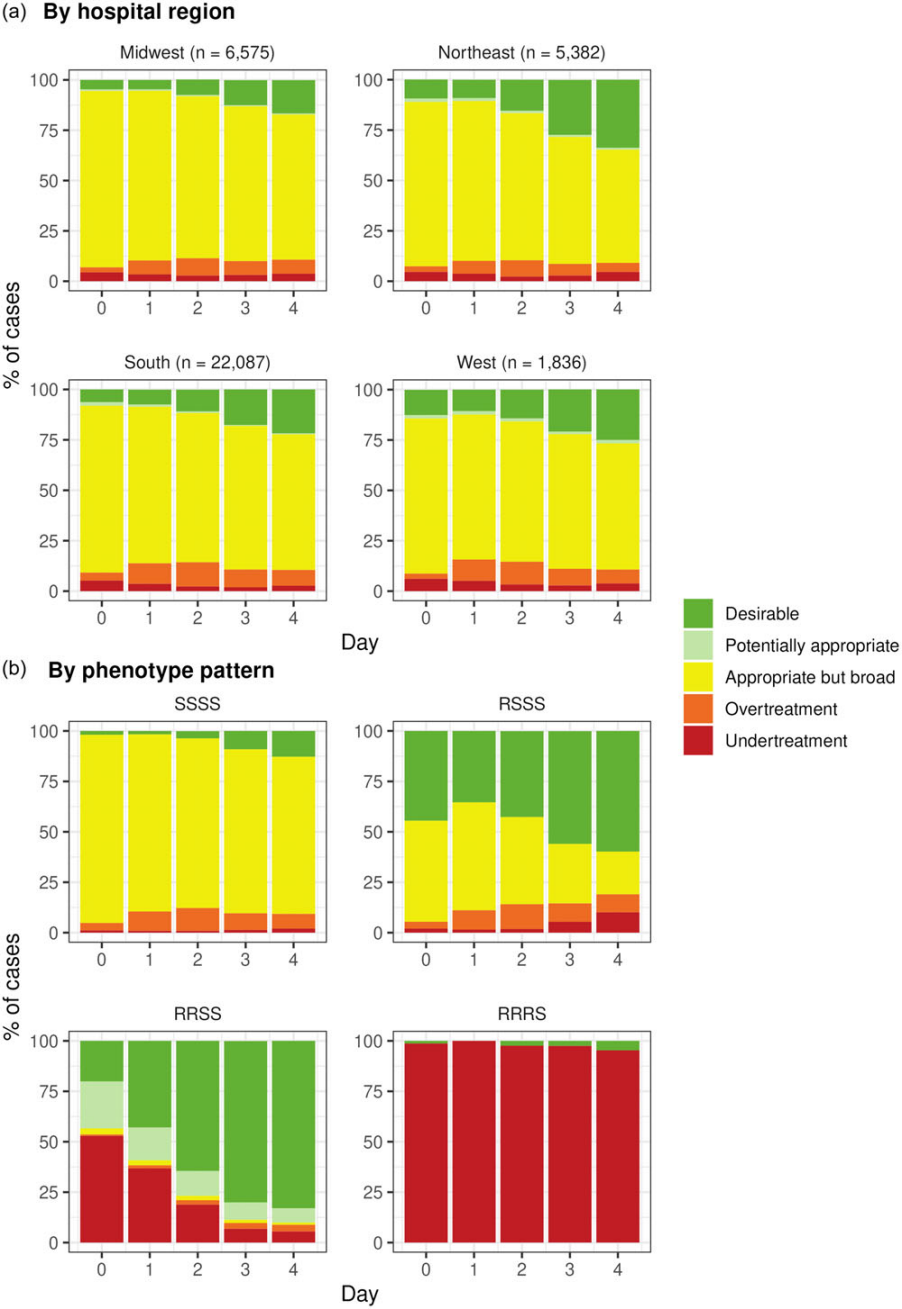
Proportion of patients receiving each category of Desirability of Outcome Ranking for the Management of Antimicrobial Therapy (DOOR-MAT) agents. (a) By hospital region. (b) By phenotype pattern.

In our study cohort, the proportion of patients receiving PDAT increased from 7% to 23% from BCC Day 0 to Day 4 (Figure 3). The proportion of patients receiving appropriate but broad treatment decreased from 83% to 66% from BCC Day 0 to Day 4. On the other hand, overtreatment initially increased from 4% to 11% from BCC Day 0 to Day 2 before decreasing to 7% by BCC Day 4. The proportion of patients receiving undertreatment was low on BCC Day 0 (5%), and it decreased to 3% by BCC Day 4. Figure 4a and 4b show the differences in the proportion of patients receiving each DOOR-MAT category by geographic regions of hospitals and final phenotypic antimicrobial susceptibility patterns, respectively.

### Patients receiving PDAT versus No PDAT

Demographic characteristics were similar between those receiving PDAT versus non-PDAT (Table 1). Patients receiving PDAT were more likely to visit a large (500+ beds, 36% vs 31%), teaching (45% vs 39%), and urban (85% vs 82%) hospitals in the Northeast (22% vs 13%) compared to non-PDAT (all *P* < .01). Presence of baseline comorbidities and prior use of antimicrobials were similar between the two groups of patients.

Most (96%) non-PDAT patients had a pathogen with S-S-S-S phenotype pattern (i.e., susceptible to all DOOR-MAT categories), while less than half (47%) of the PDAT patients did. Only a handful of non-PDAT patients had R-S-S-S (i.e., resistant to Narrow-Spectrum Penicillins/Cephalosporins category and susceptible to the rest of DOOR-MAT categories) and R-R-S-S (i.e., resistant to Narrow-Spectrum Penicillins/Cephalosporins and Intermediate I but susceptible to the rest of DOOR-MAT categories) phenotype patterns (3% and 2%, respectively), but 28% and 25% of PDAT patient did, respectively. Less than 1% of the patients had R-R-R-S (i.e., only susceptible to the Broadest category) phenotype pattern in both groups. Among PDAT patients, most common pathogen was *E. coli* (78%), followed by *K. pneumoniae* (14%) and *P. mirabilis* (7%). Slightly higher percentage of patients had *K. pneumoniae* (19%) and *P. mirabilis* (10%) among non-PDAT patients.

### Patients receiving early PDAT versus delayed PDAT

Among PDAT patients, 5,033 patients (61%) received early PDAT. Early PDAT patients were more likely to be Hispanic (10% vs 7%), slightly younger (mean age 68 vs 70 years), and less likely to have Medicare (68% vs 73%, all *P* <. 01, Table 2). They were also more likely to visit a small (1–299 beds, 40% vs 38%) rural (17% vs 13%) hospitals in the West (7% vs 5%) compared to delayed PDAT patients (all *P* < .01). Early PDAT patients had a lower mean CCI score (3.2 vs 3.6) and were less likely to have severe or extreme Severity of Illness (71% vs 79%) compared to delayed PDAT patients (all *P* < .01).

**Table 2.** Demographic, hospital, and visit and clinical characteristics of patients hospitalized with gram-negative bloodstream infection, stratified by the timing of phenotype-desirable antimicrobial therapy (PDAT) status

	Overall		Early PDAT		Delayed PDAT		*P* values
	(n = 8,193)		(n = 5,033)		(n = 3,160)		
***Patient demographics***							
**Age, continuous, in years**							
Mean-Std Dev	69.0	16.4	68.2	16.9	70.3	15.6	<0.01
**Sex, n (%)**							
Male	3,435	41.9%	2,079	41.3%	1,356	42.9%	0.15
Female	4,758	58.1%	2,954	58.7%	1,804	57.1%	
Unknown							
**Race, n (%)**	5,778	70.5%	3,549	70.5%	2,229	70.5%	<0.01
White	1,200	14.6%	677	13.5%	523	16.6%	
Black	396	4.8%	269	5.3%	127	4.0%	
Asian	703	8.6%	460	9.1%	243	7.7%	
Other	116	1.4%	78	1.5%	38	1.2%	
Unknown							
**Ethnicity, n (%)**	729	8.9%	509	10.1%	220	7.0%	<0.01
Hispanic	6,393	78.0%	3854	76.6%	2539	80.3%	
Non-Hispanic	1071	13.1%	670	13.3%	401	12.7%	
Unknown							
**Insurance type, n (%)**	5,746	70.1%	3,425	68.1%	2,321	73.4%	<0.01
Medicare	870	10.6%	571	11.3%	299	9.5%	
Medicaid	1140	13.9%	746	14.8%	394	12.5%	
Commercial insurance	273	3.3%	187	3.7%	86	2.7%	
Uninsured	164	2.0%	104	2.1%	60	1.9%	
Other/Unknown							
***Visit and Clinical Characteristics***							
**Charlson-Deyo comorbidities, n (%)**							
Myocardial infarction	1,176	14.4%	723	14.4%	453	14.3%	0.97
Congestive heart failure	2,100	25.6%	1,221	24.3%	879	27.8%	<0.01
Peripheral vascular disease	859	10.5%	466	9.3%	393	12.4%	<0.01
Cerebrovascular disease	1023	12.5%	594	11.8%	429	13.6%	0.02
Dementia	1,491	18.2%	878	17.4%	613	19.4%	0.03
Chronic pulmonary disease	2,161	26.4%	1,323	26.3%	838	26.5%	0.82
Rheumatic disease	352	4.3%	217	4.3%	135	4.3%	0.93
Peptic ulcer disease	185	2.3%	98	1.9%	87	2.8%	0.02
Mild liver disease	186	2.3%	117	2.3%	69	2.2%	0.68
Diabetes	3,697	45.1%	2,233	44.4%	1,464	46.3%	0.08
Hemiplegia or paraplegia	213	2.6%	119	2.4%	94	3.0%	0.09
Renal disease	2,627	32.1%	1,577	31.3%	1,050	33.2%	0.07
Moderate or severe liver disease	211	2.6%	121	2.4%	90	2.8%	0.22
Any malignancy	1,288	15.7%	736	14.6%	552	17.5%	<0.01
Metastatic solid tumor	444	5.4%	230	4.6%	214	6.8%	<0.01
HIV disease	36	0.4%	23	0.5%	13	0.4%	0.76
**Charlson Comorbidity Index (CCI) score**							
Mean-Std Dev	3.32	2.83	3.16	2.77	3.56	2.91	<0.01
Median (Q1-Q3)	3.00	(1.00–5.00)	2.00	(1.00–5.00)	3.00	(1.00–5.00)	
**Severity of illness (3M APR-DRG), n (%)**							
Minor	221	2.7%	161	3.2%	60	1.9%	*<0.01*
Moderate	1,877	22.9%	1278	25.4%	599	19.0%	
Severe	3,569	43.6%	2,145	42.6%	1,424	45.1%	
Extreme	2,526	30.8%	1,449	28.8%	1,077	34.1%	
**Treatment patterns, n (%)**							
S-S-S-S	3,811	46.5%	1,582	31.4%	2,229	70.5%	*<0.01*
R-S-S-S	2,323	28.4%	1,838	36.5%	485	15.3%	
R-R-S-S	2,055	25.1%	1,610	32.0%	445	14.1%	
R-R-R-S	4	0.0%	3	0.1%	1	0.0%	
**Types of infection organism, n (%)**							
*E. coli*	6,395	78.1%	4,071	80.9%	2,324	73.5%	*<0.01*
*K. oxytoca*	104	1.3%	69	1.4%	35	1.1%	
*K. pneumoniae*	1,124	13.7%	619	12.3%	505	16.0%	
*P. mirabilis*	570	7.0%	274	5.4%	296	9.4%	
**Concurrent bacterial infection, present on admission, n (%)**							
Pneumonia	809	9.9%	494	9.8%	315	10.0%	*0.82*
Urinary tract infection	6,603	80.6%	4,154	82.5%	2,449	77.5%	*<0.01*
Sepsis	6,749	82.4%	4,149	82.4%	2,600	82.3%	*0.86*
Bacteremia	833	10.2%	500	9.9%	333	10.5%	*0.38*
Central venous catheter-related infection	76	0.9%	44	0.9%	32	1.0%	*0.52*
***Hospital characteristics***							
**Hospital Size, n (%)**	3,214	39.2%	2,026	40.3%	1,188	37.6%	0.03
1–299 beds	2,029	24.8%	1204	23.9%	825	26.1%	
300–499 beds	2,950	36.0%	1,803	35.8%	1,147	36.3%	
500+ beds							
**Teaching Status, n (%)**	3,690	45.0%	2,264	45.0%	1,426	45.1%	0.90
Teaching hospital	4,503	55.0%	2,769	55.0%	1,734	54.9%	
Non-teaching hospital							
**Population Served, n (%)**	1,266	15.5%	852	16.9%	414	13.1%	<0.01
Rural	6,927	84.5%	4,181	83.1%	2,746	86.9%	
Urban							
**Geographic Location, n (%)**	1,146	14.0%	699	13.9%	447	14.1%	<0.01
Midwest	1,791	21.9%	1021	20.3%	770	24.4%	
Northeast	4,773	58.3%	2,966	58.9%	1,807	57.2%	
South	483	5.9%	347	6.9%	136	4.3%	
West	535	6.5%	366	7.3%	169	5.3%	<0.01

APR-DRG, All Patient Defined Diagnosis Related Groups; HIV, human immunodeficiency virus.

*If the patient received more than one applicable agent on the same day, all given agents were captured.

Overall, the mean time to the first AST result was 3 days. There was only a 2-hour difference in time to results between early compared to delayed PDAT patients (71.2 ± 27.7 hours versus 73.2 ± 24.9 hours, respectively). More patients in the early PDAT group had a resistant phenotype compared to delayed PDAT (54% vs 29%). A higher percentage of patients receiving early PDAT had *E. coli* infection (81% vs 74%) and presented with urinary tract infection upon admission (83% vs 78%) compared to patients receiving delayed PDAT (Table 2).

The most common first effective antimicrobial was Intermediate I (e.g., ceftriaxone, 57%), followed by Intermediate II (e.g., cefepime, piperacillin-tazobactam, 54%), Broad (e.g., carbapenems, 42%), and Broadest (e.g., ceftazidime-avibactam, 28%) in the early PDAT group. In the delayed PDAT group, the most common first effective antimicrobial was Intermediate II (72%), followed by Intermediate I (59%), Narrow-Spectrum Cephalosporins (e.g., cefazolin, 49%), and Narrow-Spectrum Penicillins (e.g., ampicillin-sulbactam, 23%). Among patients with a pathogen of R-R-S-S phenotype pattern, 29% in the early PDAT group and 34% in the delayed PDAT group received Intermediate II as the first effective antimicrobial.

### Patients receiving effective oral therapy

Patients receiving oral antimicrobials (n = 4,155, 11.6%) were more likely to be younger (mean age 67 vs 69), women (64% vs 60%), and visit a large (38% vs 31%) and teaching hospital (45% vs 40%) compared to patients not receiving effective oral therapy. Among these patients, 881 patients (21%) received early oral antimicrobials (i.e., between BCC Day 0 and 2). Patients receiving early effective oral therapy were younger (mean age 66 vs 68 years) and more likely to visit a teaching hospital (48% vs 44%) in the Midwest (24% vs 19%) compared to patients receiving delayed effective oral therapy (all *P* < .01, Supplement eTable 3). Baseline comorbidities were similar between the two groups (mean CCI score 2.9 vs 2.8, *P* = 0.31) and a lower percentage of patients receiving early effective oral therapy had a severe or extreme level of severity of illness (60% vs 67%) compared to patients receiving delayed effective oral therapy (*P* < .01). The number of patients receiving oral effective oral therapy increased from 168 (4%) on BCC Day 0–2,749 (66%) on BCC Day 4 (Supplement eFigure 1). Most frequently received effective oral agents were FQ, followed by oral β-lactams, and TMP-SMX. The most commonly used oral β-lactams were amoxicillin-clavulanate, cefpodoxime, cefuroxime, and cefdinir.

## Discussion

We used one of the largest hospital-based administrative databases in the US to examine the prevalence and characteristics of BSI patients receiving PDAT, early PDAT, and delayed PDAT. Our findings showed that the receipt of early PDAT was associated with hospital and pathogen characteristics, as well as severity of illness.

In this study, we attempted to expand the framework definition of DOOR-MAT categories to additional GN-BSI pathogens (i.e., *K. oxytoca* and *P. mirabilis*) and describe the antimicrobial use pattern in a more generalizable hospitalized population in the US in recent years compared to previous studies.^20,26^ Four pathogens were included in this study because there is a stronger agreement on appropriateness of therapy and focus on beta-lactam agents for these pathogens.^20^ Furthermore, these pathogens represented approximately two-thirds of GN-BSI in the US based on SENTRY surveillance database.^27,28^

In the study from Perez *et al.* ^20^ an improvement in desirability of definitive treatment (antibiotics received the day after AST) compared with empiric treatment (antibiotics received the day before AST results) was seen for patients with *E. coli* or *K. pneumoniae* BSI in Veterans Health Administration medical centers between 2009 and 2018. The proportion of cases receiving desirable treatment improved from ∼10% during the empiric period to ∼20% in the definitive treatment period. In our study population of patients with *E. coli*, *K. pneumoniae, K. oxytoca,* and *P. mirabilis* BSI between 2017 and 2022, we showed that the proportion of patients receiving desirable treatment increased from 7% on the date of BCC to 23% on BCC Day 4. This indicates that the improvement in more desirable antibiotic therapies between empiric and definitive treatment periods observed by Perez *et al.* ^20^ may be extended to recent years as well as to a more generalizable inpatient population in the US. Furthermore, we observed that the proportion of patients receiving overtreatment was the highest on BCC Day 2. The finding is consistent with improvement in de-escalation from broad empiric to narrower definitive therapy during an inpatient stay observed by Perez *et al.* ^20^

Additionally, Perez *et al.* ^20^ noted that the most frequently used empiric therapy was piperacillin-tazobactam, followed by ceftriaxone and ciprofloxacin for both *E. coli* and *K. pneumoniae*. We similarly observed that the antimicrobial category of the first effective therapy among patients receiving PDAT were Intermediate II (e.g., cefepime, piperacillin-tazobactam, 61%), followed by Intermediate I (e.g., ceftriaxone, cefotaxime, 58%). We would like to note that 152 patients in the delayed PDAT group received Intermediate II as the first effective antimicrobial. We categorized these patients as receiving delayed PDAT because we did not consider cefepime and piperacillin-tazobactam as desirable therapies for extended-spectrum β-lactamase-producing (ESBL) Enterobacterales. However, depending on the definition of desirable therapy for ESBL (as mixed opinions exist in the literature^29^), these patients could have been considered as receiving early PDAT.

Findings by Kadri *et al.* ^26^ showed that the discordant empirical antimicrobial therapy (i.e., undertreatment) was high (about 1 in 5) among BSI patients in the US which was not significantly affected by hospital-level characteristics. Our study population had a low proportion of patients with undertreatment (2%–5%) and PDAT patients were slightly more likely to visit a large (500+ beds), teaching, and urban hospitals in the Northeast compared to non-PDAT patients.

Although we expected the use of oral antimicrobials for BSIs to be limited to patients meeting certain criteria and some hesitancy or delay if the patient has more comorbidities, no difference was observed in pre-existing comorbid conditions between patients receiving early effective oral therapy compared to those receiving delayed effective oral therapy. However, severity of illness was lower among early effective oral therapy patients compared to delayed effective oral therapy patients.

We observed that the mean time to first AST results was approximately 3 days. While this may indicate many early PDAT patients receiving PDAT as an empiric treatment, a significant number of patients in this group also received more than 1 antibiotic agents within 2 days of BCC. There are potential patient (e.g., evolution of clinical response or deterioration) and microbiology result (e.g., notification of positive blood culture, gram stain results, molecular blood test results) factors that could have informed antibiotic decisions within 2 days of BCC. As demonstrated in the secondary analysis of RAPIDS-GN, a randomized controlled trial evaluating the clinical impact of rapid AST for GN bacteremia, availability of rapid AST methods may facilitate receipt of early PDAT for patients who do not receive desirable treatment.^30^ At the same time, we would like to note that, still, less than a quarter of the patients received PDAT by BCC Day 4. Demographic and clinical characteristics of patients receiving PDAT were similar to that of patients not receiving PDAT. This highlights the need for strategies to improve and optimize antibiotic use for patients with GN-BSI including diagnostic improvement, antimicrobial stewardship interventions, and educational efforts.

### Limitations

This study has several limitations. First, this was a secondary data analysis using a hospital administrative database. Many clinical conditions, except those defined using microbiology data, were captured by ICD-10-CM or ICD-10-PCS codes, and potential coding errors may affect the accuracy of patient identification. Furthermore, definition of ‘present on admission’ was based on hospital-reporting, which was not a requirement for reporting on some conditions. Second, the timestamp of the antimicrobial administration was not available. Therefore, the timing of antimicrobial administration in reference to BCC was based on the date (e.g., BCC Day 0–4), not time (e.g., within 24 hours). Third, we were not able to assess the use and role of RDTs. Fourth, we excluded polymicrobial and intra-abdominal infections that often require broad-spectrum antimicrobials making the categorization of desirable treatment challenging.

## Conclusions

This is one of the few studies to describe patient, hospital, and clinical characteristics of BSI patients with *E. coli*, *K. pneumoniae, K. oxytoca,* and *P. mirabilis* infection receiving early PDAT versus delayed PDAT in the US. Receipt of PDAT was associated with hospital characteristics and pathogen characteristics (including phenotype and pathogen type) and less with baseline condition of patients. Early receipt of PDAT was associated with hospital and pathogen characteristics, as well as severity of illness, and most AST results took ∼ 3 days. Further studies are needed to assess the relationship between timing of PDAT and clinical outcomes.

## Supporting information

Moon et al. supplementary material Moon et al. supplementary material

## Supplementary material

For supplementary material accompanying this paper visit http://doi.org/10.1017/ash.2024.444. click here to view supplementary material
