# Supplementary material for: Incidence and variability in receipt of phenotype-desirable antimicrobial therapy for Enterobacterales bloodstream infections among hospitalized United States patients
Source: Antimicrob Steward Healthc Epidemiol. 2024 Oct 22;4(1):e183. doi: 10.1017/ash.2024.444 (PMC11500314; doi:10.1017/ash.2024.444)
Supplement: Moon et al. supplementary material [file S2732494X24004443sup001.docx]

**Supplement**

**Manuscript Title:** Incidence and Variability in Receipt of Phenotype-Desirable Antimicrobial Therapy for *Enterobacterales* Bloodstream Infections among Hospitalized United States Patients

**Authors:** Rena C. Moon, MD, MPH; Shawn H. MacVane, PharmD; Joy David, BS; Jacob B. Morton, PharmD, MBA; Ning Rosenthal, MD, PhD, MPH; Kimberly C. Claeys, PharmD, PhD

**eTable 1. Names and categorizations of DOOR-MAT agents and oral agents**

| **Categorization** | **Antibiotics** | **Route** | **Generic and Brand Names** |
| --- | --- | --- | --- |
| Narrow-Spectrum Penicillins | Ampicillin, amoxicillin,  ampicillin-sulbactam, amoxicillin-clavulanate | Intravenous (IV), intramuscular (IM), Oral | **Ampicillin**, Totacillin-N, Omnipen-N |
|  |  |  | **Amoxicillin** |
|  |  |  | **Ampicillin-sulbactam**, Unasyn |
|  |  |  | **Amoxicillin-clavulanate**, Augmentin |
| Narrow-Spectrum Cephalosporins | 1^st^ and 2^nd^ generation cephalosporins | IV, IM, Oral | **Cefazolin**, Ancef, Kefzol |
|  |  |  | **Cephalexin**, Keflex, Biocef, Zartan, Penixine, Daxbia |
|  |  |  | **Cefadroxil**, Duricef |
|  |  |  | **Cefprozil**, Cefzil |
|  |  |  | **Cefuroxime**, Ceftin, Kefurox, Zinacef |
|  |  |  | **Cefoxitin**, Mefoxin |
|  |  |  | **Cefotetan**, Cefotan |
|  |  |  | **Cefaclor**, Ceclor, Raniclor |
| Oral 3^rd^ Generation Cephalosporins | 3^rd^ generation cephalosporins | Oral | **Cefdinir**, Omnicef |
|  |  |  | **Cefixime**, Suprax |
|  |  |  | **Cefpodoxime**, Vantin |
|  |  |  | **Cefditoren**, Spectracef |
|  |  |  | **Ceftibuten**, Cedax |
| Intermediate I | 3^rd^ generation cephalosporins | IV, IM | **Ceftriaxone**, Rocephin |
|  |  |  | **Cefotaxime**, Claforan |
|  |  |  | **Ceftaroline**, Teflaro |
| Intermediate II | Cefepime, piperacillin-tazobactam, ceftazidime | IV, IM | **Cefepime**, Maxipime |
|  |  |  | **Ceftazidime**, Fortax, Tazicef, Ceptaz, Tazidime |
|  |  |  | **Piperacillin-tazobactam**, Zosyn |
| Broad | Carbapenems | IV, IM | **Ertapenem**, INVanz |
|  |  |  | **Imipenem-cilastatin**, Primaxin |
|  |  |  | **Doripenem**, Doribax |
|  |  |  | **Meropenem**, Merrem |
| Broadest | New β-lactam/β-lactam inhibitors (Ceftazidime-avibactam) | IV, IM | **Ceftazidime-avibactam**, Avycaz |
|  |  |  | **Ceftolozane-tazobactam**, Zerbaxa |
|  |  |  | **Meropenem-vaborbactam**, Vabomere |
|  |  |  | **Imipenem-relebactam**, Recarbrio |
|  |  |  | **Cefiderocol**, Fetroja |
| Fluoroquinolones | Fluoroquinolones | Oral | **Ciprofloxacin**, Cipro |
|  |  |  | **Levofloxacin**, Levaquin |
|  |  |  | **Moxifloxacin**, Avelox |
|  |  |  | **Delafloxacin,** Baxdela |
| Trimethoprim-sulfamethoxazole | Trimethoprim-sulfamethoxazole | Oral | **TMP-SMX**, Bactrim, Sufatrim, Septra, Sulfatrim, Bethaprim, Cotrim, Uroplus |

**eTable 2. Charlson-Deyo comorbidities and related ICD-10-CM diagnosis and procedure codes**

| **Comorbidity** | **Type** | **Deyo’s Algorithm with ICD-10-CM codes** |
| --- | --- | --- |
| Myocardial infarction | Dx | I21.x, I22.x, I23.x, I25.2 |
| Congestive heart failure | Dx | I50.x |
| Peripheral vascular disease | Dx | I73.9, I71.00, I71.01, I71.02, I71.03, I71.1, I71.2, I71.3, I71.4, I71.5, I71.6, I71.8, I71.9, I96, Z95.828 |
|  | Proc | 04RK07Z, 04RK0JZ, 04RK0KZ, 04RK47Z, 04RK4JZ, 04RK4KZ, 04RL07Z, 04RL0JZ, 04RL0KZ, 04RL47Z, 04RL4JZ, 04RL4KZ, 04RM07Z, 04RM0JZ, 04RM0KZ, 04RM47Z, 04RM4JZ, 04RM4KZ, 04RN07Z, 04RN0JZ, 04RN0KZ, 04RN47Z, 04RN4JZ, 04RN4KZ, 04RP07Z, 04RP0JZ, 04RP0KZ, 04RP47Z, 04RP4JZ, 04RP4KZ, 04RQ07Z, 04RQ0JZ, 04RQ0KZ, 04RQ47Z, 04RQ4JZ, 04RQ4KZ, 04RR07Z, 04RR0JZ, 04RR0KZ, 04RR47Z, 04RR4JZ, 04RR4KZ, 04RS07Z, 04RS0JZ, 04RS0KZ, 04RS47Z, 04RS4JZ, 04RS4KZ, 04RT07Z, 04RT0JZ, 04RT0KZ, 04RT47Z, 04RT4JZ, 04RT4KZ, 04RU07Z, 04RU0JZ, 04RU0KZ, 04RU47Z, 04RU4JZ, 04RU4KZ, 04RV07Z, 04RV0JZ, 04RV0KZ, 04RV47Z, 04RV4JZ, 04RV4KZ, 04RW07Z, 04RW0JZ, 04RW0KZ, 04RW47Z, 04RW4JZ, 04RW4KZ, 04RY07Z, 04RY0JZ, 04RY0KZ, 04RY47Z, 04RY4JZ, 04RY4KZ |
| Cerebrovascular disease | Dx | I60.x, I61.x, I62.x, I63.x, I65.x, I66.x, I67.x, I68.x, I69.x, G45.x |
| Dementia | Dx | F03.90, F01.50, F01.51, F03.91, F02.80, F02.81 |
| Chronic pulmonary disease | Dx | J40, J41.0, J41.1, J44.9, J44.0, J41.8, J42, J43.9, J45.20, J45.21, J45.22, J44.1, J45.990, J45.991, J45.909, J45.998, J45.902, J45.901, J47.9, J47.1, J67.0, J67.1, J67.2, J67.3, J67.4, J67.5, J67.6, J67.7, J67.8, J67.9, J60, J61, J62.8, J63.0, J63.1, J63.2, J63.3, J63.4, J63.5, J63.6, J66.0, J66.1, J66.2, J66.8, J64, J68.4 |
| Rheumatic disease | Dx | M32.10, M34.0, M34.1, M34.9, M33.20, M06.9, M05.00, M05.30, M05.60, M06.1, M05.10, M35.3 |
| Peptic ulcer disease | Dx | K25.0, K25.1, K25.2, K25.3, K25.4, K25.5, K25.6, K25.7, K25.9, K26.0, K26.1, K26.2, K26.3, K26.4, K26.5, K26.6, K26.7, K26.9, K27.0, K27.1, K27.2, K27.3, K27.4, K27.5, K27.6, K27.7, K27.9, K28.0, K28.1, K28.2, K28.3, K28.4, K28.5, K28.6, K28.7, K28.9 |
| Mild liver disease | Dx | K70.30, K73.9, K73.0, K75.4, K73.2, K73.8, K74.0, K74.60, K74.69, K74.3, K74.4, K74.5 |
| Diabetes without chronic complication | Dx | E11.9, E10.9, E13.9, E11.65, E10.65, E13.65, E10.1x, E11.1x, E13.1x, E11.0x, E13.0x, E11.64x, E10.64x, E13.64x |
| Diabetes with chronic complication | Dx | E10.2x, E10.3x, E10.4x, E10.5x, E10.61x, E10.62x, E10.63x, E10.69, E10.8, E11.2x, E11.3x, E11.4x, E11.5x, E11.61x, E11.62x, E11.63x, E11.69, E11.8, E13.2x, E13.3x, E13.4x, E13.5x, E13.61x, E13.62x, E13.63x, E13.69, E13.8 |
| Hemiplegia or paraplegia | Dx | G04.1, G11.4, G80.1, G80.2, G81.x,  G82.x, G83.0, G83.1x, G83.2x, G83.3x, G83.4, G83.9 |
| Moderate or severe renal disease | Dx | I12.0, I13.11, I13.2, N03.2, N03.3, N03.5, N03.8, N03.9, N05.2 N05.5, N05.9, N08, N18.x, N19.x, N25.x, Z49.0x, Z49.3x, Z94.0, Z99.2 |
| Any malignancy, including lymphoma and leukemia, except malignant neoplasm of skin | Dx | C00.x-C75.x (except C43.x and C44.x), C81.x-C85.x, C88.x, C90.x, C91.x-C95.x, C96.x, C7A.xxx, C7B.xxx, D00.xx, D01.xx, D02.x, D03.xx, D05.xx, D06.x, D07.xx, D09.xx, D47.9 |
| Moderate or severe liver disease | Dx | I85.00, I85.01, I85.10, I85.11, I86.4, K70.4x, K71.1x, K71.7, K72.xx, K76.6, K76.7 |
| Metastatic solid tumor | Dx | C77.x, C78.x, C79.x, C80.0 |
| HIV disease | Dx | B20 |

Dx: diagnosis

Proc: procedure

HIV: human immunodeficiency virus

**eTable 3**. **Demographic, hospital, and visit and clinical characteristics of patients hospitalized with gram-negative bloodstream infection, stratified by the timing of effective oral therapy^a^**

|  | **Overall** | | **Early Effective Oral Therapy** | | **Delayed Effective Oral Therapy** | | **p-values** |
| --- | --- | --- | --- | --- | --- | --- | --- |
|  | **(N = 4,155)** | | **(n = 881)** | | **(n = 3,274)** | |  |
| ***Patient demographics*** |  |  |  |  |  |  |  |
| **Age, continuous, in years** |  |  |  |  |  |  |  |
| Mean-Std Dev | 67.1 | 17.3 | 65.8 | 18.5 | 67.5 | 16.9 | *0.01* |
| **Sex, n (%)** |  |  |  |  |  |  |  |
| Male | 1,511 | 36.4% | 296 | 33.6% | 1,215 | 37.1% | *0.05* |
| Female | 2,644 | 63.6% | 585 | 66.4% | 2,059 | 62.9% |  |
| Unknown | 0 | 0.0% | 0 | 0.0% | 0 | 0.0% |  |
| **Race, n (%)** |  |  |  |  |  |  |  |
| White | 3,070 | 73.9% | 655 | 74.3% | 2,415 | 73.8% | *0.59* |
| Black | 588 | 14.2% | 131 | 14.9% | 457 | 14.0% |  |
| Asian | 168 | 4.0% | 32 | 3.6% | 136 | 4.2% |  |
| Other | 271 | 6.5% | 49 | 5.6% | 222 | 6.8% |  |
| Unknown | 58 | 1.4% | 14 | 1.6% | 44 | 1.3% |  |
| **Ethnicity, n (%)** |  |  |  |  |  |  |  |
| Hispanic | 376 | 9.0% | 70 | 7.9% | 306 | 9.3% | *0.27* |
| Non-Hispanic | 3,308 | 79.6% | 718 | 81.5% | 2,590 | 79.1% |  |
| Unknown | 471 | 11.3% | 93 | 10.6% | 378 | 11.5% |  |
| **Insurance type, n (%)** |  |  |  |  |  |  |  |
| Medicare | 2,772 | 66.7% | 577 | 65.5% | 2,195 | 67.0% | *0.25* |
| Medicaid | 414 | 10.0% | 101 | 11.5% | 313 | 9.6% |  |
| Commercial insurance | 705 | 17.0% | 148 | 16.8% | 557 | 17.0% |  |
| Uninsured | 199 | 4.8% | 46 | 5.2% | 153 | 4.7% |  |
| Other/Unknown | 65 | 1.6% | 9 | 1.0% | 56 | 1.7% |  |
| ***Clinical characteristics*** |  |  |  |  |  |  |  |
| **Charlson-Deyo comorbidities, n (%)** |  |  |  |  |  |  |  |
| Myocardial infarction | 514 | 12.4% | 104 | 11.8% | 410 | 12.5% | *0.57* |
| Congestive heart failure | 836 | 20.1% | 177 | 20.1% | 659 | 20.1% | *0.98* |
| Peripheral vascular disease | 378 | 9.1% | 71 | 8.1% | 307 | 9.4% | *0.23* |
| Cerebrovascular disease | 476 | 11.5% | 102 | 11.6% | 374 | 11.4% | *0.90* |
| Dementia | 565 | 13.6% | 117 | 13.3% | 448 | 13.7% | *0.76* |
| Chronic pulmonary disease | 1,046 | 25.2% | 232 | 26.3% | 814 | 24.9% | *0.37* |
| Rheumatic disease | 174 | 4.2% | 35 | 4.0% | 139 | 4.2% | *0.72* |
| Peptic ulcer disease | 77 | 1.9% | 17 | 1.9% | 60 | 1.8% | *0.85* |
| Mild liver disease | 83 | 2.0% | 17 | 1.9% | 66 | 2.0% | *0.87* |
| Diabetes | 1,736 | 41.8% | 351 | 39.8% | 1,385 | 42.3% | *0.19* |
| Hemiplegia or paraplegia | 77 | 1.9% | 13 | 1.5% | 64 | 2.0% | *0.35* |
| Renal disease | 1,102 | 26.5% | 231 | 26.2% | 871 | 26.6% | *0.82* |
| Moderate or severe liver disease | 87 | 2.1% | 19 | 2.2% | 68 | 2.1% | *0.88* |
| Any malignancy | 636 | 15.3% | 120 | 13.6% | 516 | 15.8% | *0.12* |
| Metastatic solid tumor | 259 | 6.2% | 51 | 5.8% | 208 | 6.4% | *0.54* |
| HIV disease | 23 | 0.6% | 9 | 1.0% | 14 | 0.4% | *0.03* |
| **Charlson Comorbidity Index (CCI) score,** |  |  |  |  |  |  |  |
| Mean-Std Dev | 2.99 | 2.81 | 2.90 | 2.80 | 3.01 | 2.82 | *0.31* |
| Median (Q1-Q3) | 2.00 | (1.00- 4.00) | 2.00 | (1.00- 4.00) | 2.00 | (1.00- 4.00) |  |
| **Severity of illness (3M APR-DRG), n (%)** |  |  |  |  |  |  |  |
| Minor | 193 | 4.6% | 56 | 6.4% | 137 | 4.2% | *< 0.01* |
| Moderate | 1,287 | 31.0% | 299 | 33.9% | 988 | 30.2% |  |
| Severe | 1,923 | 46.3% | 364 | 41.3% | 1,559 | 47.6% |  |
| Extreme | 752 | 18.1% | 162 | 18.4% | 590 | 18.0% |  |
| **Types of infection organism, n (%)** |  |  |  |  |  |  |  |
| *E. coli* | 3,045 | 73.3% | 657 | 74.6% | 2,388 | 72.9% | *0.43* |
| *K. oxytoca* | 81 | 1.9% | 21 | 2.4% | 60 | 1.8% |  |
| *K. pneumoniae* | 762 | 18.3% | 152 | 17.3% | 610 | 18.6% |  |
| *P. mirabilis* | 267 | 6.4% | 51 | 5.8% | 216 | 6.6% |  |
| **Acute kidney injury, present on admission, n (%)** | 1,783 | 42.9% | 320 | 36.3% | 1,463 | 44.7% | *< 0.01* |
| **Concurrent bacterial infection, present on admission, n (%)** |  |  |  |  |  |  |  |
| Pneumonia | 375 | 9.0% | 78 | 8.9% | 297 | 9.1% | *0.84* |
| Urinary tract infection | 3,364 | 81.0% | 709 | 80.5% | 2,655 | 81.1% | *0.68* |
| Sepsis | 3,293 | 79.3% | 648 | 73.6% | 2,645 | 80.8% | *< 0.01* |
| Bacteremia | 509 | 12.3% | 118 | 13.4% | 391 | 11.9% | *0.24* |
| Central venous catheter-related infection | 40 | 1.0% | 13 | 1.5% | 27 | 0.8% | *0.08* |
| ***Hospital characteristics*** |  |  |  |  |  |  |  |
| **Hospital Size, n (%)** |  |  |  |  |  |  |  |
| 1-299 beds | 1,579 | 38.0% | 339 | 38.5% | 1,240 | 37.9% | *0.07* |
| 300-499 beds | 1,013 | 24.4% | 190 | 21.6% | 823 | 25.1% |  |
| 500+ beds | 1,563 | 37.6% | 352 | 40.0% | 1,211 | 37.0% |  |
| **Teaching Status, n (%)** |  |  |  |  |  |  |  |
| Teaching hospital | 1,851 | 44.5% | 423 | 48.0% | 1,428 | 43.6% | *0.02* |
| Non-teaching hospital | 2,304 | 55.5% | 458 | 52.0% | 1,846 | 56.4% |  |
| **Population Served, n (%)** |  |  |  |  |  |  |  |
| Rural | 701 | 16.9% | 145 | 16.5% | 556 | 17.0% | *0.71* |
| Urban | 3,454 | 83.1% | 736 | 83.5% | 2,718 | 83.0% |  |
| **Geographic Location, n (%)** |  |  |  |  |  |  |  |
| Midwest | 827 | 19.9% | 208 | 23.6% | 619 | 18.9% | *< 0.01* |
| Northeast | 703 | 16.9% | 170 | 19.3% | 533 | 16.3% |  |
| South | 2,426 | 58.4% | 467 | 53.0% | 1,959 | 59.8% |  |
| West | 199 | 4.8% | 36 | 4.1% | 163 | 5.0% |  |

APR-DRG: All Patient Defined Diagnosis Related Groups; HIV: human immunodeficiency virus

^a^Receiving effective oral therapy was identified if the patient received any of the oral treatment in eTable 1 that the pathogen was susceptible to within 4 days of blood culture collection date. Among patients receiving effective oral therapy, the patient was identified as ‘receiving early effective oral therapy’ if effective oral therapy was given within 2 days of BCC, and ‘receiving delayed effective oral therapy’ otherwise.

**eFigure 1. Number of patients receiving effective oral therapy and composition of effective oral therapy agents between blood culture collection days 0-4**


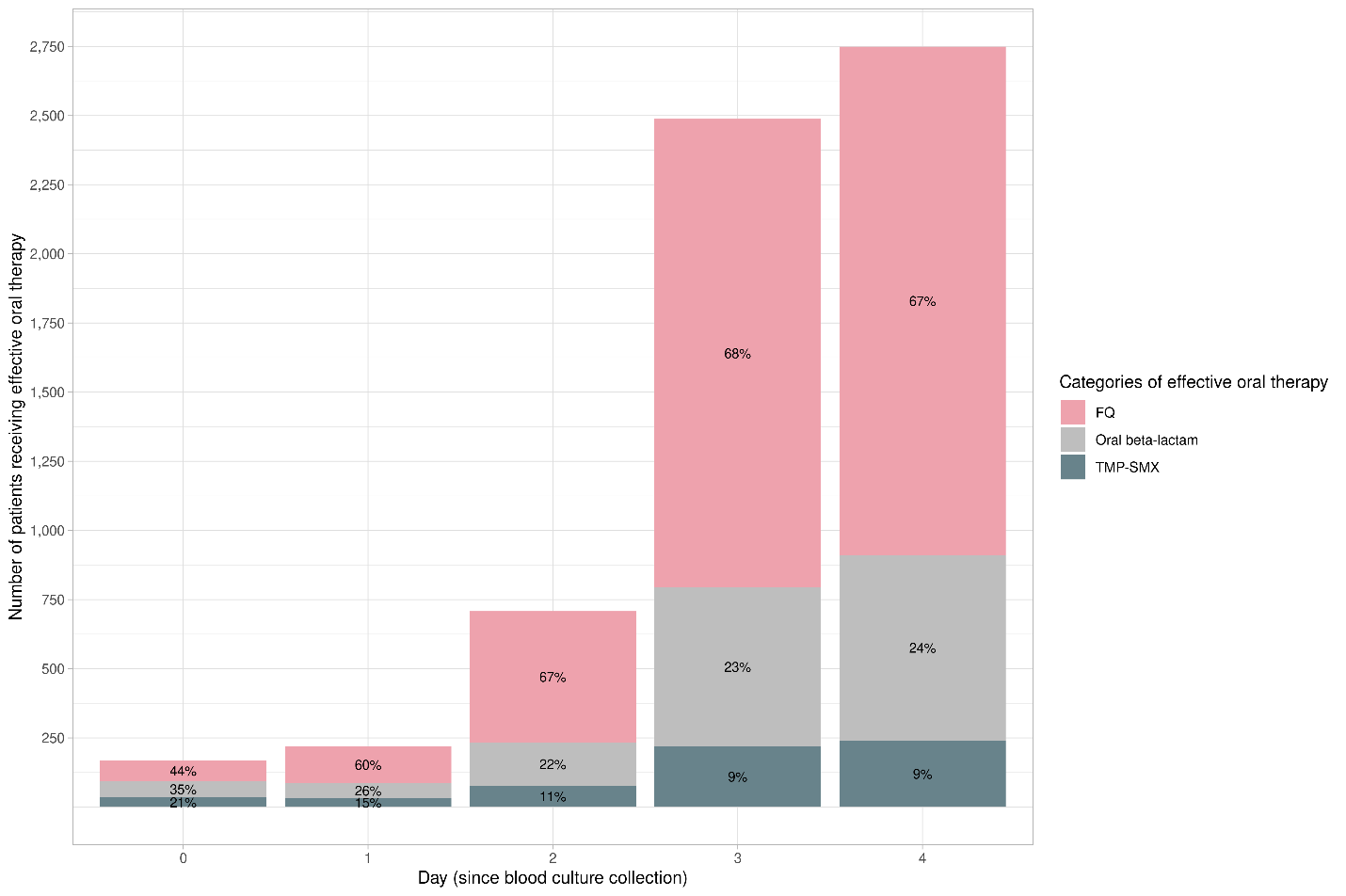


FQ: fluoroquinolone; TMP-SMX: trimethoprim-sulfamethoxazole
